# Current and Future Therapies for Pancreatic Ductal Adenocarcinoma

**DOI:** 10.3390/cancers14102417

**Published:** 2022-05-13

**Authors:** Áine Sally, Ryan McGowan, Karen Finn, Brian Michael Moran

**Affiliations:** 1 Department of Analytical, Biopharmaceutical and Medical Sciences, School of Science and Computing, Atlantic Technological University Galway City, Dublin Road, H91 T8NW Galway, Ireland; aine.sally@research.gmit.ie (Á.S.); ryan.mcgowan@mail.itsligo.ie (R.M.); karen.finn@gmit.ie (K.F.); 2 Department of Life Sciences, School of Science, Atlantic Technological University Sligo, Ash Lane, Ballytivnan, F91 YW50 Sligo, Ireland

**Keywords:** pancreatic ductal adenocarcinoma, chemotherapy, chemoresistance, immunotherapy, synthetic lethality, clinical trials

## Abstract

**Simple Summary:**

Pancreatic cancer is the fourth leading cause of cancer-related mortality worldwide. The poor survival associated with this disease is due to delayed diagnosis, a lack of reliable biomarkers, and tumour resistance to treatment. Currently, surgery is the only curative treatment option, but few patients are eligible for this procedure. Developing resistance to current chemotherapies such as gemcitabine has led to a reduction in effective therapy options for patients and an urgent requirement for the development of novel therapeutic avenues. Potential success has been noted in therapeutic approaches such as synthetic lethality and immunotherapy. An array of clinical trials are currently recruiting, primarily in the area of monoclonal antibodies in combination with other therapies such as chemotherapy and immune checkpoint inhibitors. This review article aims to highlight the potential these therapies have to improve patient prognosis and survival.

**Abstract:**

Pancreatic cancer is one of the leading causes of cancer-related death worldwide. This is due to delayed diagnosis and resistance to traditional chemotherapy. Delayed diagnosis is often due to the broad range of non-specific symptoms that are associated with the disease. Resistance to current chemotherapies, such as gemcitabine, develops due to genetic mutations that are either intrinsic or acquired. This has resulted in poor patient prognosis and, therefore, justifies the requirement for new targeted therapies. A synthetic lethality approach, that targets specific loss-of-function mutations in cancer cells, has shown great potential in pancreatic ductal adenocarcinoma (PDAC). Immunotherapies have also yielded promising results in the development of new treatment options, with several currently undergoing clinical trials. The utilisation of monoclonal antibodies, immune checkpoint inhibitors, adoptive cell transfer, and vaccines have shown success in several neoplasms such as breast cancer and B-cell malignancies and, therefore, could hold the same potential in PDAC treatment. These therapeutic strategies could have the potential to be at the forefront of pancreatic cancer therapy in the future. This review focuses on currently approved therapies for PDAC, the challenges associated with them, and future directions of therapy including synthetically lethal approaches, immunotherapy, and current clinical trials.

## 1. Introduction

Pancreatic cancer is the fourth leading cause of cancer-related mortality worldwide, with a five-year survival rate of less than 8% [1]. It can be classed into endocrine and exocrine tumours with PDAC, a form of exocrine pancreatic cancer, the most common form [2]. PDAC accounts for approximately 90% of all pancreatic cancer cases. Obesity, smoking, and type 2 diabetes mellitus are modifiable risk factors linked to the development of PDAC [3]. Approximately 5–6% of all PDAC cases have germline mutations that pose further risk to the patient [1]. Genetic mutations detected in PDAC are most commonly associated with the oncogene *KRAS* and tumour suppressor genes *CDKN2A*, *TP53,* and *SMAD4*/*DPC4* [4]. The genotypic profile of each of these mutations and the number of different mutations present can vary widely between patients. At least one or more of these mutations can be detected in PDAC and, therefore, could harbour the potential for future therapeutic strategies such as synthetic lethality [5].

The high mortality rate associated with PDAC is primarily due to delayed diagnosis and tumour resistance to chemotherapy [2]. A broad range of non-specific symptoms are associated with PDAC such as abdominal pain, jaundice, dry/itchy skin, steatorrhoea, and bilirubinuria. This could lead to a delayed diagnosis, as 43% of staged cases present at stage IV [6]. This delay in diagnosis can have an impact on treatment options for patients, as currently, treatment depends on the stage of the tumour. For resectable cases, surgery is the primary treatment option, with chemotherapy being administered as adjuvant therapy in select cases. However, very few PDAC cases are eligible for surgery, approximately 10% to 20% [7]. This is due to delayed diagnosis and, hence, further disease progression. For unresectable cases, chemotherapy can be administered as mono- or combination therapy depending on the patient’s stage and toleration of treatment [8]. Resistance has been noted amongst chemotherapeutic strategies, most notably in gemcitabine—the first-line chemotherapeutic drug for PDAC [9]. Though it is the cornerstone of PDAC treatment, it yields a meagre efficacy of approximately 20% to 30%, with most patients acquiring resistance of unknown mechanisms [9].

Resistance to chemotherapy has become a critical problem in the treatment of PDAC, with most patients displaying resistance patterns [10]. Chemoresistance can be categorised into intrinsic or acquired resistance. Intrinsic resistance is apparent at the start of treatment due to genetic factors unique to the patient which can cause modifications to drug transport mechanisms, metabolism, and apoptotic pathways [11]. PDAC patients are not screened prior to initiating treatment due to genetic heterogeneity and instability associated with the disease [12]. Conversely, acquired resistance develops after a period of time depending on the tumour and treatment. Exposure of the tumour cells to the drug leads to genetic or epigenetic modifications within the tumour cells which ultimately leads to impaired treatment efficacy [4].

One form of intrinsic resistance that is also a pivotal characteristic of PDAC is the dense tumour microenvironment (TME) [13]. Encapsulation of the tumour in dense fibrous scar tissue, also called stromal desmoplasia, is central to intrinsic PDAC treatment resistance [4]. This stroma is made up of several components that influence cancer progression in PDAC. These include a myriad of cell types (tumour cells, cancer-associated fibroblasts, macrophages, pancreatic stellate cells, and immune cells), their products (for example interleukin-1, interleukin-6, and transforming growth factor-β), and a network of blood vessels in a rich extracellular matrix (including collagen, fibronectin, and hyaluronan) which accounts for 90% of the total tumour volume [4,14,15]. This encasement in hyperplastic connective tissue distorts the pancreatic structure leading to the development of fibrous hyperplasia [14]. This also puts stress on local blood vessels and weakens the perfusion of oxygen and vital nutrients to the surrounding tissue, resulting in hypoxia [11]. The exact cellular and molecular mechanisms of stroma-mediated intrinsic chemoresistance are yet unknown [11]. The dense TME associated with PDAC also plays a role in tumour resistance to immunotherapeutic strategies [16]. PDAC tumours can be classified into “hot” or “cold” tumour environments, with a high or low infiltration of T cells, respectively. The use of immunotherapy has yielded success with “hot” tumour environments; however, resistance has been noted as a major challenge with “cold” PDAC tumour environments and the use of immunotherapies [17,18,19]. The challenges associated with PDAC treatment today have led to therapies evolving to become more personalised to the patient [20]. The development of personalised medicine involves tailoring a patient’s treatment regime to best suit the characteristics of their malignancy and may provide a new treatment avenue for patients [20]. This review outlines therapies employed in the treatment of PDAC today, the obstacles that are currently being faced, and potential future therapies together with current recruiting clinical trials.

## 2. Current Approved Therapies

### Chemotherapy

**Figure 1:**
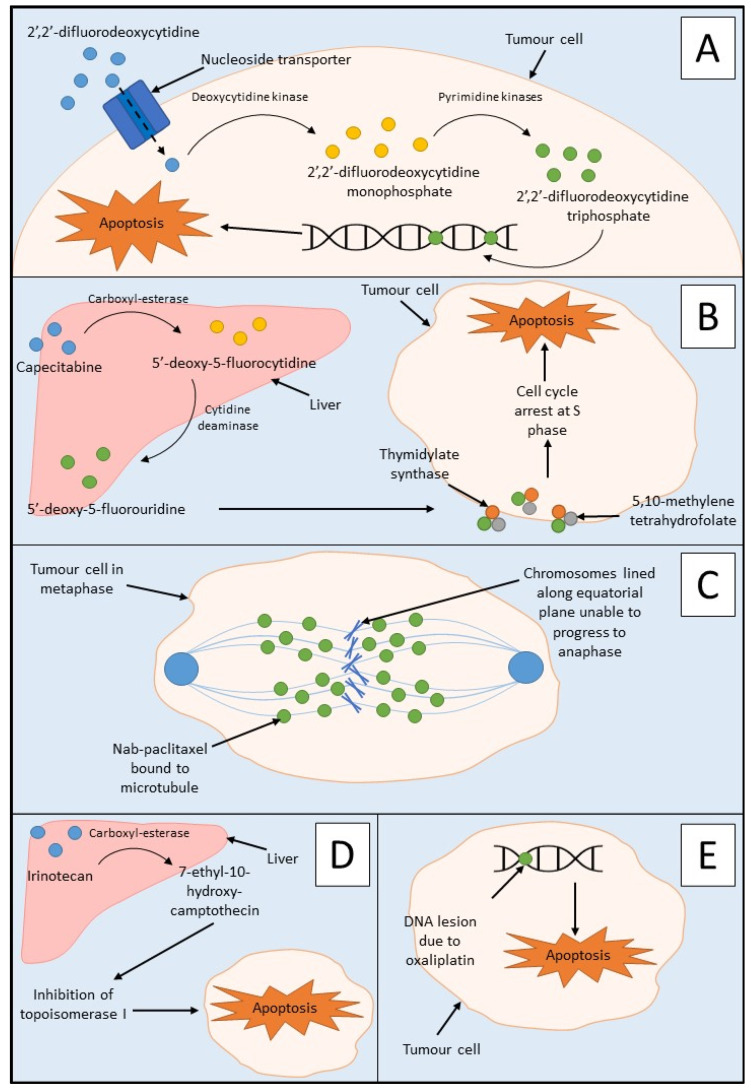
Mechanism of action of PDAC chemotherapies: (**A**) the mechanism of action of gemcitabine showing administration of prodrug 2′,2′-difluorodeoxycytidine, conversion to 2′,2′-difluorodeoxycytidine monophosphate, and 2′,2′-difluorodeoxycytidine triphosphate, respectively leading to incorporation into DNA and apoptosis of the tumour cell; (**B**) mechanism of action of capecitabine showing its metabolism to 5′-deoxy-5-fluorocytidine by carboxyl-esterase in the liver. This is further metabolised to 5′-deoxy-5′-fluorouridine by cytidine deaminase in both the liver and the tumour. 5′-deoxy-5′-fluorouridine inhibits thymidylate synthase by forming a ternary complex with thymidylate synthase and 5,10-methylenetetrahydrofolate. The formation of thymidine is stopped by the inhibition of thymidylate synthase, and therefore, DNA synthesis is blocked in S phase of the cell cycle’ (**C**) mechanism of action of nab-paclitaxel showing the binding of paclitaxel to the tubulin beta-subunit of the microtubules. This leads to the inability of the chromosomes to separate resulting in the inhibition of mitosis of the tumour cell and inevitably apoptosis; (**D**) mechanism of action of irinotecan through administration of the camptothecin-derivative prodrug. Carboxyl-esterase in the liver converts irinotecan to its active form, 7-ethyl-10-hydroxy-camptothecin, which causes impaired DNA synthesis via the inhibition of topoisomerase I preventing the removal of torsional stress. This then leads to a double-stranded DNA break, ultimately leading to cell death; (**E**) mechanism of action of oxaliplatin showing the induction of apoptosis of tumour cells due to DNA damage via DNA lesions which leads to the inhibition of both DNA and messenger RNA.

The treatment guidelines for PDAC that are the focus of this review were developed by the National Institute for Health and Care Excellence (NICE). These guidelines describe in detail the treatment strategies for all stages of PDAC [8]. Currently, chemotherapy is the main form of treatment for unresectable PDAC tumours [21]. More commonly known as gemcitabine, 2′,2′-difluorodeoxycytidine is an antimetabolite drug that inhibits DNA synthesis during the S phase of the cell cycle (Figure 1A) [22]. Gemcitabine is utilised as mono- or combination therapy (with capecitabine or albumin-bound nanoparticle paclitaxel (nab-paclitaxel)) in the treatment strategies of several stages of PDAC including locally advanced PDAC, metastatic PDAC, and as adjuvant therapy in resectable cases [8]. Treatment with gemcitabine alone yields dismal results with progression-free survival (PFS) of 3.9 months (95% CI, 3.0–5.1) and overall survival (OS) of 9.2 months (95% CI, 8.3–10.4) [23]. 

Capecitabine is an antimetabolite chemotherapeutic agent utilised in the treatment of PDAC [24]. It is administered as a pre-prodrug of 5-fluorouracil and is, therefore, an antimetabolite drug which inhibits thymidylate synthase (Figure 1B) [24,25]. Capecitabine may be used in combination with gemcitabine as adjuvant therapy in resectable PDAC if tolerated by the patient and may also be used in combination with radiotherapy for locally advanced unresectable PDAC cases [8]. A combination of stereotactic body radiation therapy, gemcitabine, and capecitabine yielded a PFS of 12 months (95% CI, 8.34–15.66) and an OS of 19 months (95% CI, 14.6–23.4) [26].

Nab-paclitaxel is another drug that is utilised in the treatment of metastatic PDAC in combination with gemcitabine if FOLFIRNOX is not tolerated by the patient [8]. Paclitaxel is a chemotherapeutic agent that causes cell death by interfering with microtubule function during mitosis (Figure 1C) [27]. Combination treatment of gemcitabine and nab-paclitaxel yielded a PFS with a median value of 6.7 months (95% CI, 6.0–8.0) and a median OS of 10 months (95% CI, 7.9–12.1) [28].

Another form of chemotherapy, FOLFIRINOX, is a combination therapy consisting of 5-fluorouracil, folinic acid, oxaliplatin, and irinotecan, and is used in the first-line treatment of metastatic PDAC [8,29]. Furthermore, 5-fluorourcail is an antimetabolite drug which inhibits thymidylate synthase, and this reaction is stabilised by folinic acid [25]. The mechanism of action of 5-fluorouracil is similar to that of capecitabine in the latter stages (from 5′-deoxy-5-fluorouradine), as seen in Figure 1B. Irinotecan is a derivative of camptothecin which causes impaired DNA synthesis via the inhibition of topoisomerase I (Figure 1D) [29]. Oxaliplatin is an alkylating chemotherapeutic drug which induces apoptosis of tumour cells due to DNA damage via DNA lesions, leading to the inhibition of both DNA and messenger RNA (Figure 1E) [29]. FOLFIRINOX remains a valuable treatment option for PDAC, with a median PFS of 13.6 months (95% CI, 11.3–15.9) and a median OS of 35.4 months (95% CI, 23.8–45.0) [30].

## 3. Targeted Therapeutic Approaches

### 3.1. Synthetic Lethality

**Figure 2:**
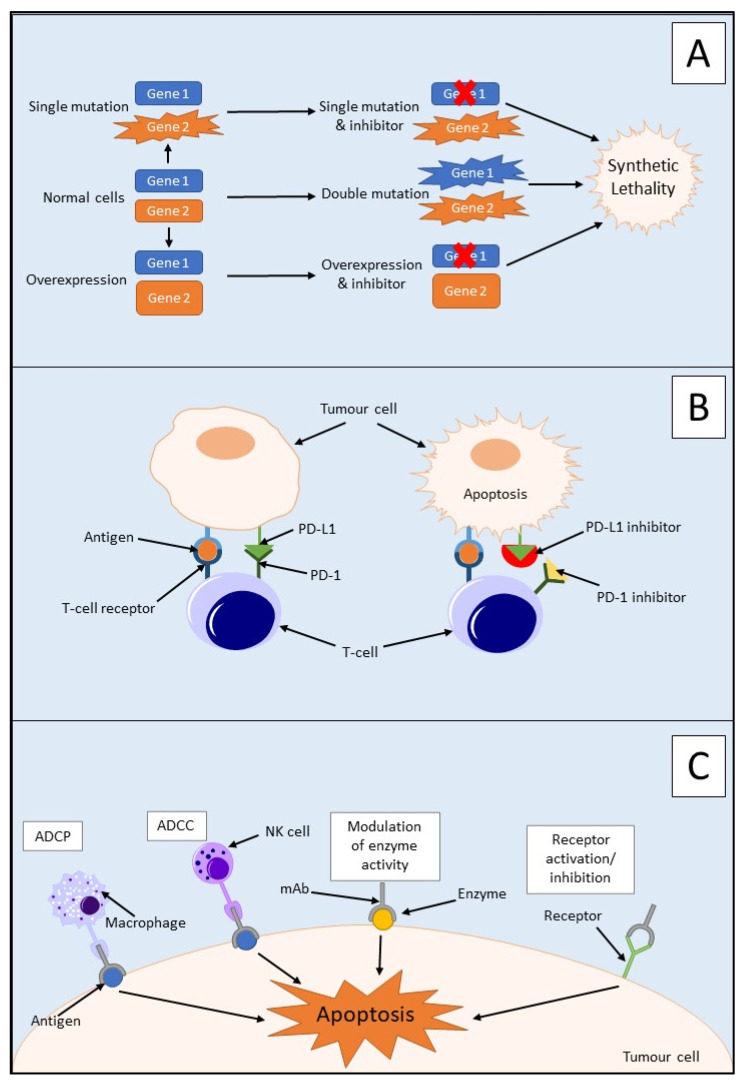
Mechanism of action of three PDAC immunotherapies: (**A**) the mechanism of synthetic lethality showing normal cells, single mutation, or overexpression of one gene is viable for a cell but inhibition of one gene and mutation in the other, a double mutation, or overexpression of one gene and inhibition of the other leads to cell death via cell viability; (**B**) mechanism of action of immune checkpoint inhibitors, for example, PD-1 and PD-L1. Binding of PD-1 and PD-L1 inhibits activation of T cells. Upregulation of both PD-1 and PD-L1 in tumour cells makes them an ideal target for inhibition leading to the activation of T cells; (**C**) mechanism of action of monoclonal antibodies (mAbs) in cancer treatment. mAbs may be used to target different molecules in the treatment of cancer, for example, receptors, antigens, or enzymes. Receptors on macrophages may target Fc portions of antigen-bound mAb and engulf tumour cells through antibody-dependent cellular phagocytosis (ADCP). Receptors on natural killer (NK) cells may also target Fc portions of antigen-bound mAb and imitate antibody-dependent cellular cytotoxicity (ADCC).

Synthetic lethality is used to define an interaction between two genes in which a mutation of one is viable; however, a mutation of both leads to cell death, as outlined in Figure 2A [31,32]. It can also involve a mutation only being synthetically lethal if it is combined with another specific mutation. These synthetically lethal interactions can be described as gain-of-function alleles or loss-of-function alleles, with the most common being the latter [33]. Loss-of-function alleles can be categorised based on their protein product′s function; for example, they could have an essential function, be a subunit of a protein complex, or play a role in protein folding pathways. Synthetic lethality has proven successful in the treatment of *BRCA1/2*-mutated neoplasms which are sensitive to poly(ADP-ribose) polymerase (PARP) inhibitors [34]. PARP is an enzyme which is involved in the repair of single-strand DNA breaks (SSBs) [32]. Inhibition of PARP will lead to irreparable SSBs and, therefore, a one-ended double-stranded DNA break (DSB) via the collapse of a replication fork. Neoplasms containing a loss-of-function mutation of either the *BRCA1* or *BRCA2* gene lack homologous recombination; therefore, DSB results in cell death [32]. Due to this aspect, synthetic lethality is believed to be a possible route for potential anticancer drugs in the treatment of PDAC via the proteins produced by potential synthetically lethal genes with mutations [33]. However, commonly occurring loss-of-function mutations in PDAC such as those in *CDKN2A*, *TP53*, and *SMAD4* are currently not targetable due to the many genetic aberrations, mainly point mutations, associated with each gene [35].

The success yielded from *BRCA1/2* and PARP inhibitors could provide insight into other potential treatment routes to be explored for PDAC, in particular, DNA damage repair pathways [34]. In PDAC, somatic mutations often occur in DNA damage repair pathway genes, for example, *TP53* (68.90%), *BRCA2* (4.40%), and *ATM* (4.00%) [36]. These mutations could result in synthetically lethal targets being utilised alongside chemotherapeutic agents to enhance the effect of treatment. Clustered regularly interspaced short palindromic repeat (CRISPR) screens have been used to identify potential synthetically lethal mutations via gene knockout screening in vivo using an orthotopic patient-derived xenograft mouse model [37]. It was found that downregulation of protein arginine methyltransferase gene 5 (*PRMT5*) enhances gemcitabine sensitivity in PDAC cells [37]. This gene encodes an enzyme that is responsible for the methylation of arginine residues on proteins such as histones and transcription factors and, therefore, plays a pivotal role in the cell cycle and transcription. The inhibition of this enzyme halts both DNA repair and DNA synthesis leading to increased tumour cell apoptosis. Combination treatment of gemcitabine with PRMT5 inhibition provides a synergistic treatment pair that may provide a synthetically lethal combination for the treatment of PDAC in the future [37].

### 3.2. Immunotherapy

With the issue of growing resistance to current PDAC treatments, new and more targeted therapies are required to improve the prognosis of patients. As previously mentioned, the TME plays a major role in intrinsic resistance to chemotherapeutic drugs. Immunotherapy is the use of treatments and therapies that target and stimulate an immune response to combat cancer [38]. There are several different forms of immunotherapy available such as the use of immune checkpoint inhibitors, monoclonal antibodies, adoptive cell transfer, and vaccination. Research into the use of these immunotherapeutic strategies for several different solid tumours, including PDAC, has increased over recent decades, due to its outstanding success in the past [38]. With approximately 25% of breast cancer cases positive for human epithelial growth receptor 2 (HER2), the monoclonal antibody trastuzumab has forged a new path in the treatment of this malignancy [39]. Another example is rituximab, an anti-CD20 monoclonal antibody that is a cornerstone drug in the treatment of B-cell malignancies such as non-Hodgkin lymphoma and chronic lymphocytic leukaemia [40].

#### 3.2.1. Immune Checkpoint Inhibitors

Immune checkpoint inhibitors are an immunotherapeutic strategy that activates the immune system to modulate the immune response to cancer [41]. This occurs through the stimulation of the innate and adaptive immune systems, with the main focus on the activation of T cells, as outlined in Figure 2B. Although this has proven useful for other tumours, for example, the use of nivolumab and pembrolizumab has aided in the survival of malignant melanoma patients, it has not been the case for PDAC [42,43]. PDAC has previously displayed resistance towards immune checkpoint inhibitors which could be mainly due to the dense TME present. The central cause of this resistance is the presence of myeloid cell populations within PDAC tumours including monocytes, macrophages, and granulocytes [43,44]. This immune-privileged TME within the tumours can cause T-cell dysfunction via mechanisms such as T-cell anergy leading to immunosuppression [44].

Programmed death-1 (PD-1) is an immune checkpoint protein that is activated by its ligand PD-L1 and is expressed by activated T cells [45]. PD-L1 is a membrane protein that is expressed on immune and tumour cells and inactivates T cells by inducing programmed cell death via heterodimer formation with CD80 [46,47]. Overexpression of PD-L1 has been detected in several cancers, including PDAC, and this overexpression is associated with advanced tumour stage and, therefore, a poorer prognosis for patients [45,48]. PD-L1 blockade alone has been shown to display minimal inhibition in PDAC and, therefore, is not regarded as a sufficient therapeutic target alone. This could be due to the non-immunogenic nature of PDAC or immunosuppression due to the high tumour burden [45].

Due to the poor success of immune checkpoint inhibitor monotherapy in PDAC, potential combination therapies are being sought to improve its efficacy. Cancer Forkhead box protein 3 (cFOXP3) is an upregulated protein in PDAC and plays a vital role with regulatory T cells and immune evasion in cancer [49]. It achieves this through the recruitment of cFOXP3 positive regulatory T cells to the site of malignancy via upregulation of C–C chemokine ligand 5 (CCL5). PD-L1 expression in PDAC has been shown to coexist with regulatory T-cell infiltration of tumours. This suggests a possible link between PD-L1 and cFOXP3 expression [49]. cFOXP3 has been shown to upregulate PD-L1 in mouse cells, primary human PDAC cells, and PDAC cell lines (MIA PaCa-2 and AsPC-1) [38]. It was also shown that cFOXP3 induces PD-L1 inhibition of cytotoxic T-cell activity and, therefore, plays a role in immune evasion in PDAC [49].

Inhibition of CCL5 alone in PDAC has shown dismal results in pre-clinical trials and is not enough to reduce PDAC progression; however, combination therapy of anti-CCL5 and anti-PD-L1 has shown potential therapeutic effects in PDAC via enhanced interferon-gamma secretion and significantly upregulating tumour infiltrating CD8+ T cells (*p* < 0.05 and *p* < 0.01) [46]. The combination of both anti-CCL5 and anti-PD-L1 inhibited tumour growth, in both a cFOXP3 Pan02 xenograft model and an orthotopic murine model, via the stimulation of cytotoxic T cells. A significantly higher OS of approximately 35 days was obtained with the combination of anti-CCL5 and anti-PD-L1, compared with approximate OS values of 27 days and 30 days for anti-PD-L1 and anti-CCL5 monotherapy, respectively, in the orthotopic murine model (*p* < 0.01). This combination therapy could be a potential treatment option for PDAC patients in the future, with one of its variations used in currently recruiting clinical trials [46,50].

#### 3.2.2. Monoclonal Antibodies

The four main mechanisms of action of monoclonal antibodies are outlined in Figure 2C. There are several studies on monoclonal antibodies that could potentially bring new targeted therapies to the forefront of PDAC treatment and improve patient prognosis [5]. In a study investigating monoclonal antibody pairs and their effect on monolayer BxPC-3 cells and CD1 nude mice injected with BxPC-3 cells, it was found that two strategies worked to combat PDAC in this animal model [5]. The first strategy was the combination of two antibodies which were both specific to either epidermal growth factor receptor (EGFR) or HER2. This was described as a homo-combination of antibodies. The second was the combination of antibodies consisting of a pair of antibodies, one to EGFR and the other to HER2. This was described as a hetero-combination of antibodies. The homo-combination of antibodies yielded better results than the hetero-combinations at carrying out anticancer effects via the degradation of their respective receptor [5].

Cytotoxic T-lymphocyte-associated antigen 4 (CTLA-4) is an immune checkpoint protein which is expressed on T cells, in particular regulatory T cells [51]. Ligands for CTLA-4 include CD80 and CD86, both molecules are expressed on antigen-presenting cells. The use of anti-CTLA-4 antibody ipilimumab in advanced PDAC has not yielded much success [52]. However, blockade of CTLA-4 with anti-CTLA-4 in the KrasG12D/+; Trp53R172H/+; Pdx-1Cre (KPC) PDAC mouse model displayed infiltration of the tumour with CD4+ T cells [51]. This study suggested that this tumour infiltration with CD4+ T cells occurred via CTLA-4 expressed on regulatory T cells interacting with CD80-expressing dendritic cells present in peritumoral lymph nodes, thus demonstrating a role for CTLA-4/CD80 interaction in T-cell exclusion. The combined use of anti-interleukin-6 and anti-CTLA-4 was found to promote antitumour activity via enhancement of T-cell tumour infiltration [53]. Significantly increased tumour inhibition was observed in mice receiving the combination treatment of antibodies, compared with mice receiving monotherapy of the specific antibodies (CTLA-4 *p* = 0.0207, interleukin-6 *p* = 0.0002) [53].

Another example of the potential use of monoclonal antibodies in PDAC was seen in a study using anti-CD47 as adjuvant therapy in resectable PDAC [54]. CD47 is a transmembrane protein which is highly expressed in some tumour cells, including PDAC [54,55]. This allows the cells to evade phagocytosis by macrophages, and this could be a mechanism for micrometastasis, the spread of a small number of tumour cells to another organ. Murine models were established by injecting athymic nude (Foxn1^nu^) or NOD *scid* gamma (NOD.*Cg-Prkdc^scid^Il2rg^tm1Wjl^*/SzJ), with prepared primary PDAC tumour samples to determine the effects of CD47 blockade. The natural ability of hepatic macrophages to protect against micrometastases was enhanced via increased engulfment of tumour cells observed in vitro and in vivo. Mice treated with anti-CD47 showed significantly increased PFS, with obtained *p*-values of <0.0001 and 0.002. These mice also showed significantly increased OS with an obtained *p*-value of 0.002. Anti-CD47 shows potential for a new adjuvant therapy for PDAC to improve treatment efficacy and, therefore, the prognosis of patients [54].

#### 3.2.3. Adoptive Cell Transfer

Adoptive cell transfer is the transfer of immune cells into a patient as a form of therapy to improve the patient’s immune system. The use of natural killer cells as a form of adoptive cell transfer therapy has recently become a point of interest in the field of immunotherapy [56]. This is due to their ability to target and eliminate tumour cells via cytotoxic mechanisms and the role they play in inducing an adaptive immune response. In the pre-cancerous stages of PDAC, there is a loss of natural killer cells due to mutations in *KRAS*. This could be due to their involvement in the initiation and progression of PDAC. A murine KPC model of PDAC was used to determine the effect of donor natural killer cell adoptive cell transfer. There was increased necrosis of tumour cells detected in mice treated with natural killer cells along with prolonged survival (56.0 days), compared with control mice (26.5 days) with no statistical significance noted (*p* = 0.2324). This is the only study showing promise with adoptive cell transfer and PDAC with natural killer cell adoptive cell transfer, proving a potential treatment option for PDAC in the future [56].

#### 3.2.4. Therapeutic Vaccination

Any tumour cells remaining post-surgery/treatment could lead to a relapse of the PDAC tumour [57]. Therefore, vaccination yields a possible approach to target remnant tumour cells via activation of the immune system toward tumour-associated antigens. The whole tumour cell lysate was utilised by processing murine PDAC tumour membranes to enable them to be opsonised by naturally occurring human IgG antibodies [57]. This can stimulate the immune system to target tumour associated antigens, in this case, galactose-alpha-1,3-galactose (α-gal). An immune response was mounted against the PDAC tumour lysate vaccine, resulting in antitumour properties in murine models. A statistically significant median OS was observed with mice treated with PDAC tumour lysate and α-gal of 95.0 days (95% CI, 69–95), compared with the untreated control mice of 40.0 days (95% CI, 35–45) (*p* < 0.01). This shows a potential route of treatment for PDAC which could be utilised in the future [57]. Another study targeted tumour-associated antigens by employing autologous murine-induced pluripotent stem cells (iPSCs) alongside the adjuvant CpG ODN1826 [58]. The use of this iPSC vaccination decreased CD4 + CD25 + FOXP3 + regulatory T cells in the murine model, thus reversing the immunosuppression of the TME while preventing the development of tumours in 75% of mice [58].

### 3.3. Ongoing Clinical Trials

At present, there are a plethora of clinical trials recruiting patients that involve the treatment of PDAC. These studies can be seen in Table 1 and display many possibilities for the treatment of PDAC in the future. Many of these studies involve the utilisation of immunotherapies, in particular, monoclonal antibodies in combination with another form of therapy such as conventional chemotherapy. An example of this is a study currently recruiting in the Cancer Centre at Johns Hopkins University which aims to determine the effects of pembrolizumab which could be given in combination with defactinib in patients with resectable PDAC as a form of neoadjuvant or adjuvant therapy [50]. Pembrolizumab is a monoclonal antibody directed towards PD-1, and this may or may not be administered intravenously alongside defactinib, a focal adhesion kinase inhibitor. Focal adhesion kinase is a non-receptor tyrosine kinase that is involved in cell scaffolding and signalling at the sites of integrin clustering on the cell membrane [59]. The effects of these drugs will be determined in combination with standard neoadjuvant and adjuvant chemotherapy regimens such as gemcitabine [50]. This study will determine if the tumour microenvironment can be reprogrammed via targeting focal adhesion kinase post-chemotherapy and, therefore, potentiate anti-PD-1 effects to halt cancer progression [50].

A separate study that is currently recruiting in the Washington School of Medicine is also utilising monoclonal antibody therapy in PDAC but is focusing on administering a combination of drugs alongside standard chemotherapy, such as gemcitabine, to see how the tumour responds [60]. BMS-813160 is a dual CCR2/CCR5 antagonist which inhibits the activation of signal transduction pathways which can be involved in inflammation and tumour cell migration, proliferation, and invasion [61]. It will be administered alongside nivolumab, a monoclonal antibody which is directed to PD-1. Chemotherapeutic agents gemcitabine and nab-paclitaxel will also be administered in combination with these therapies. This will be carried out with a cohort of patients with borderline resectable and locally advanced PDAC [60].

There have been several studies with PDAC and the use of KRAS-based vaccines as a potential treatment [62,63]. The Cancer Centre at Johns Hopkins is currently in phase 1 of a trial utilising a pooled KRAS peptide vaccine with polyinosinic–polycytidylic acid adjuvant in combination with two monoclonal antibodies: ipilimumab and nivolumab [63]. As previously mentioned, ipilimumab is a monoclonal antibody that is directed towards the immune checkpoint inhibitor CTLA-4, and nivolumab is a monoclonal antibody directed to PD-1. Interestingly, in a separate study at the University of Pennsylvania, patients with resected PDAC are currently being recruited for phase 1 of the trial determining the effects of a mature dendritic cell-based vaccine [62]. Patients will receive a vaccine produced from autologous dendritic cells pulsed with mutant KRAS peptides corresponding to each patient’s specific tumour mutation and human leukocyte antigen type. Patients will receive a primer dose, followed by a booster dose 8 weeks later along with regular monitoring of patient immune response [62]. Another trial is utilising both BMS-813160 and nivolumab as potential neoadjuvant and adjuvant therapies in locally advanced PDAC in the Cancer Centre at Johns Hopkins [64]. This study, however, is investigating the use of GVAX, a whole tumour cell vaccine which has been genetically modified to secrete granulocyte–macrophage colony-stimulating factor. The aim of this study is to determine if this combination therapy increases the infiltration of immune cells into PDAC tumours, in particular, CD8 + CD137 + cells [64].

## 4. Conclusions

PDAC remains one of the most aggressive forms of cancer worldwide, with a high cancer-related mortality rate, a five-year survival of less than 8%, and increased chemoresistance observed leading to a poorer prognosis for patients. Due to this, there is a need for more advanced therapeutic strategies to overcome resistance and improve patient prognosis. A potential therapeutic avenue that has shown promising success in PDAC is immunotherapy. The combination use of both immune checkpoint inhibitors and monoclonal antibodies has yielded the most promising results thus far in PDAC; however, there are still many obstacles to overcome. The potential of immunotherapy as a therapeutic strategy for PDAC is dictated by the presence of intrinsic resistance from the TME and “cold” tumour environment. Adoptive cell transfer and vaccination may also provide potential future therapeutic strategies for PDAC; however, at present their use remains elusive. The use of synthetic lethality has demonstrated promise in breast cancer and may provide more druggable targets for PDAC in the future. Research in PDAC treatment is increasing in recent years and will hopefully lead to better, more targeted therapies for patients in the future.

## Figures and Tables

**Table 1:** Summary table of discussed clinical trials investigating novel therapeutics for PDAC.

Treatment	Target	Stage	Phase	Reference
Pembrolizumab Defactinib	PD-1 monoclonal antibody Focal adhesion kinase inhibitor	Resectable PDAC	Recruiting, Phase 2	[50]
Nivolumab BMS-813160 GVAX	PD-1 monoclonal antibody CCR2/CCR5 dual antagonist Whole tumour cell vaccine	Locally advanced PDAC	Recruiting, Phase 1/2	[64]
BMS-813160 Nivolumab Gemcitabine Nab-paclitaxel	CCR2/CCR5 dual antagonist PD-1 monoclonal antibody Chemotherapy Chemotherapy	Borderline resectable/locally advanced PDAC	Recruiting, Phase 1/2	[60]
mDC3/8-KRAS vaccine	Mutant KRAS	Resectable PDAC	Recruiting, Phase 1	[62]
KRAS peptide vaccine Nivolumab Ipilimumab	KRAS peptide vaccine PD-1 monoclonal antibody CTLA-4 monoclonal antibody	Resected PDAC	Recruiting, Phase 1	[63]
